# How Do Women and Men Look at the Past? Large Scanpath in Women during Autobiographical Retrieval—A Preliminary Study

**DOI:** 10.3390/brainsci13030439

**Published:** 2023-03-03

**Authors:** Mohamad El Haj, Claire Boutoleau-Bretonnière, Lina Guerrero Sastoque, Quentin Lenoble, Ahmed A. Moustafa, Guillaume Chapelet, Elisa Sarda, André Ndobo

**Affiliations:** 1 Laboratoire de Psychologie des Pays de la Loire, Nantes Université, 44000 Nantes, France; 2 Centre Hospitalier Universitaire in Nantes, Clinical Gerontology Department, Bd Jacques Monod, 44093 Nantes, France; 3 Institut Universitaire de France, 75000 Paris, France; 4 Centre Hospitalier Universitaire in Nantes, Inserm Centre d’Investigation Clinique, 44000 Nantes, France; 5 Inserm, Centre Hospitalier Universitaire in Lille, Lille Neuroscience & Cognition, University of Lille, 59000 Lille, France; 6 School of Psychology, Faculty of Society and Design, Bond University, Gold Coast, QLD 4229, Australia; 7 Department of Human Anatomy and Physiology, The Faculty of Health Sciences, University of Johannesburg, Johannesburg 2006, South Africa

**Keywords:** autobiographical memory, eye movement, gender, gender differences

## Abstract

While research has consistently demonstrated how autobiographical memory triggers visual exploration, prior studies did not investigate gender differences in this domain. We thus compared eye movement between women and men while performing an autobiographical retrieval task. We invited 35 women and 35 men to retrieve autobiographical memories while their gaze was monitored by an eye tracker. We further investigated gender differences in eye movement and autobiographical specificity, that is, the ability to retrieve detailed memories. The analysis demonstrated shorter fixations, larger duration and amplitude of saccades, and higher autobiographical specificity in women than in men. The significant gender differences in eye movement disappeared after controlling for autobiographical specificity. When retrieving autobiographical memory, female participants generated a large scan with short fixation and high saccade amplitude, while male participants increased their fixation duration and showed poorer gaze scan. The large saccades in women during autobiographical retrieval may constitute an exploratory gaze behavior enabling better autobiographical memory functioning, which is reflected by the larger number of autobiographical details retrieved compared to men.

## 1. Introduction

Gender differences have been reported in cognitive functioning [1,2,3], especially regarding visual perception [4,5]. Given that biological and neural processes of visual perception can vary between males and females [4,5], different gaze patterns or visual exploration behavioral differences could be observed between women and men. Based on this hypothesis, we investigated gender differences in eye movement during autobiographical retrieval. As explained below, prior research has demonstrated how the retrieval of autobiographical memory can trigger eye movement and even how this eye movement can influence autobiographical retrieval. However, prior research has not assessed gender differences in this domain.

Compared to other memory systems, autobiographical memory is key for understanding the intersection between memory, cognition, and social interactions. While autobiographical memory supports the self [6], it also supports directive and social functions, as the retrieval of past personal experiences simulates and guides future behavior [7,8,9]. Beyond this directive function, autobiographical memory has a social goal, as the retrieval of past personal experiences allows us to communicate these experiences [10] and promotes intimacy [11,12,13]. Autobiographical memory can thus be defined as a dynamic system supporting personal memories as constructed to serve a given personal, social, and/or directive goal [14,15].

Little is known about how autobiographical retrieval can activate eye movement. One exception is a study by El Haj, Delerue [16], who recorded fixations and saccades while participants retrieved autobiographical memories and during a control condition in which participants counted aloud. Their analysis showed longer amplitudes and longer durations of saccades under the autobiographical memory condition than under the control condition. According to El Haj, Delerue [16], these eye movements reflect the attempts by the visual system to reconstruct the visual scene of the retrieved memories. Similar results were reported by El Haj, Nandrino [17], who instructed participants to remember neutral and emotional memories. Their results demonstrated fewer fixations and saccades but longer fixation duration in the retrieval of neutral memories than in the retrieval of emotional memories. Other research has investigated differences in eye movement between autobiographical retrieval and future thinking [18], demonstrating how future thinking triggers fewer fixations and saccades than past thinking. Eye movement during autobiographical retrieval may also differ depending on the temporal distribution of memories, as demonstrated by a study showing that the retrieval of remote autobiographical memories activates fewer, but longer, fixations compared to the retrieval of recent memories [19]. While eye movement can be influenced by the characteristics of autobiographical memories, the reverse pattern has also been reported, as research has demonstrated how eye movement can influence autobiographical retrieval. The latter issue was investigated by Lenoble, Janssen [20] who asked participants to remember autobiographical events while fixating on a cross on a screen or while exploring the screen without constraints. Their results demonstrated more detailed and faster memories when retrieved in the free-gaze condition than when retrieved in the maintained fixation condition. These studies show how retrieval of autobiographical memory can activate fixations and saccades and how these eye movements may mirror the generation and manipulation of mental representations in the visual system [21]. Thus, eye movements as activated by autobiographical retrieval would reflect mental imagery operations underlying autobiographical information recollection.

While the above-mentioned studies have shown how the retrieval of autobiographical memory can trigger eye movement, the impact of gender on these eye movement patterns has not yet, to our knowledge, been explored. The present study addresses this issue by comparing fixations and saccades between women and men during autobiographical retrieval. Besides being inspired by research on eye movement as activated by autobiographical memory, the present study is inspired by research demonstrating gender differences in visual processing.

One of the research topics in gender differences and eye movement is the well-known eye-tracking research demonstrating that compared to women, men tend to fixate significantly earlier and longer on women’s breasts during the visual processing of pictures depicting women’s bodies, regardless of whether the women are fully clothed [22], wearing bathing suits [23], or nude [24]. Other research has uncovered important gender differences regarding gaze, such as that women demonstrate more visual exploratory scanning strategy than men. This finding is supported by the study of Coutrot, Binetti [25], who assessed the dynamics of gaze when participants watched videos of another person. Their results demonstrated much more exploratory gaze in women than men. A similar finding was made by Gomez, von Gunten [26], who demonstrated that when viewing pictures depicting neutral and emotional scenes, women demonstrated a more exploratory scanning behavior compared to men. In a similar vein, Heisz, Pottruff [27] reported higher scanning behavior in the encoding of faces in women than in men, and that this higher scanning behavior was associated with high recognition memory in women. High explorative gaze in women was also reported by Sammaknejad, Pouretemad [28]. They analyzed regions of interest when women and men processed pictures of faces. Their analysis demonstrated that women showed a significant increase in transitions (i.e., saccades) from the regions of interest to the eyes. High explorative gaze behavior in women was also reported by Abdi Sargezeh, Tavakoli [29], who observed that, compared to women, men demonstrated less explorative gaze, as mirrored by smaller saccade amplitudes and slower scan paths, when exploring indoor scenes (e.g., a living room). Taken together, prior research has demonstrated that, compared to men, women tend to demonstrate more exploratory scanning behavior. Accordingly, in the current study, this exploratory scan pattern will, for the first time, be examined for autobiographical retrieval.

To summarize, while research has demonstrated how autobiographical memory can trigger visual exploration [17,18,20,21,30,31], this prior research has not investigated gender differences. Because gender differences have been observed for visual processing [22,24], we investigated gender differences in eye movement during autobiographical retrieval. Accordingly, we recorded fixations and saccades in women and men during autobiographical retrieval. In light of research reporting how women show high explorative gaze behavior [25,26,28,29], we predicted that we would observe a similar behavior during autobiographical retrieval. More specifically, we expected that compared to men, women would demonstrate larger saccades when retrieving autobiographical memories, such that women may tend to explore/scan the retrieved memories more than men. We further assessed the specificity of memories, in order to investigate whether gender differences in eye movement as activated by autobiographical retrieval would be influenced by the specificity of memories.

## 2. Method

### 2.1. Participants

The current study included seventy undergraduate/graduate students at the University of Nantes. Thirty-five participants were female (*M* age = 20.46 years, *SD* = 3.01, *M* education = 13.21 years, *SD* = 4.21) and thirty-five were male (*M* age = 20.98 years, *SD* = 3.14, *M* education = 13.54 years, *SD* = 4.44). No significant differences were observed between the two groups regarding age [*t*(68) = 0.73, *p* = 0.46] or educational level [*t*(68) = 0.32, *p* = 0.75].

We estimated the sample size using G*Power [32] for independent *t*-tests because our protocol involved two-group comparisons (i.e., women vs. men). This estimation involved 95% power, a probability of making Type I error of 0.05, and a large effect size of 0.80 [33]. This calculation suggested that 35 participants would be necessary in each of the two groups to obtain sufficient statistical power, i.e., a total sample size of 70 participants. Note, however, that we initially recruited a larger sample of 87 participants, from which 17 participants were excluded for several reasons, described below. The protocol was validated by the ethics committee of the University of Nantes (reference IORG0011023).

Because our study included a verbal assessment of autobiographical memory, the general verbal memory performances of participants were controlled for using the test of Grober and Buschke [34], on which participants must recall as many previously studied words as possible, with the maximum score being 16 points. The mean score of women was 12.89 (*SD* = 2.73) and that of men was 10.46 (*SD* = 2.83) [*t*(68) = 3.65, *p* = 0.001]. Note that we excluded four subjects from the original sample (*n* = 87) because their scores were two standard deviations below the expected range for their age. We excluded six other participants as they declared a history of psychiatric and/or neurological disorders. We also excluded seven participants due to difficulties with eye movement processing, including failures with the calibration of one participant and recording quality below 70% for six other participants.

### 2.2. Procedures

#### Autobiographical Memory and Eye Movement Recording

We instructed participants to verbally retrieve three autobiographical memories while wearing eye-tracking glasses and looking at a white wall. We explained that the events had to be experienced and that the events had to be specific and precise (e.g., involving spatiotemporal details). A time of two minutes was allocated to each of the three autobiographical events. No significant differences were observed between women (*M* = 87087.14 msec, *SD* = 4143.947) and men (*M* = 83108.14 msec, *SD* = 27119.93) regarding the duration of description of memories [*t*(68) = 0.64, *p* = 0.52]. Note that the instructions were repeated for each of the three memories and that, for the three memories, the participants were free to retrieve any event. We thus did not provide cue words; we rather used the same instructions for the three memories to ensure that the memories were triggered with the same instructions.

We invited the participants to remember the memory out loud while wearing eye-tracking glasses. These remote pupil-tracking glasses (Pupil Lab) had a gaze position accuracy of <0.1° and 200 Hertz sampling rate. During the experiment, we closed the blinds and kept the lightness of the room (60-watt fluorescent lamp) constant to avoid variations in retinal illumination.

### 2.3. Eye Movement Variables

The variables were the number of fixations (i.e., total number of fixations per minute), mean duration of fixations in milliseconds, number of saccades (i.e., total number of saccades per minute), mean duration of saccades in milliseconds, average amplitude of saccades (i.e., the average angle covered by saccades), and total amplitude size of saccades (i.e., the total angle of the saccades). Note that, for each variable, we considered the mean score for the three memories. Regarding blinks, we excluded them once the horizontal deviation of gaze exceeded 2° (5.5% of our dataset).

#### Autobiographical Memory Analysis

To control for whether potential gender differences in eye movement are influenced by the specificity of retrieval (e.g., whether women may retrieve more specific memories than men), we analyzed autobiographical specificity using a procedure proposed by Piolino, Desgranges [35]. For each event, zero was attributed when no memory was provided or when only general information was given about a theme; one point was attributed when an extended or a repeated event was provided; two points were attributed when events described memories with spatiotemporal details; three points were attributed when events referred to those lasting less than 24 h and situated in time and space; and four points were attributed when events referred to specific ones with spatiotemporal detail and with phenomenological information (e.g., emotion). We retained the mean scores of the three memories with a maximum score of four points.

## 3. Results

We compared the number of fixations, duration of fixations, number of saccades, duration of saccades, average and total saccade amplitude, and autobiographical specificity between women and men using independent *t*-tests. To investigate whether any significant gender differences in eye movement were influenced by autobiographical specificity, we carried out an analysis of covariance (ANCOVA) with eye movement as the dependent variable, gender as the independent variable, and autobiographical specificity as the covariate variable.

### 3.1. Shorter Fixations but Larger Saccades in Women Than in Men

**Figure 1:**
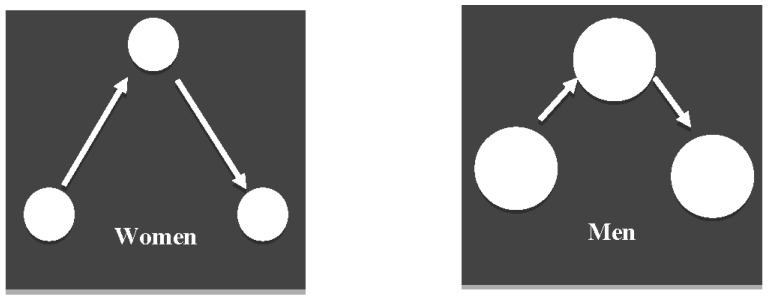
A schematic illustration of gaze during autobiographical retrieval: women demonstrated shorter fixations (i.e., smaller circles) but longer saccades (i.e., longer arrows) compared to men.

Data are provided in Table 1. The analyses demonstrated no significant effect of gender differences regarding the number of fixations [*t*(68) = 0.68, *p* = 0.50, Cohen’s *d* = 0.16] or the number of saccades [*t*(68) = 0.56, *p* = 0.58, Cohen’s *d* = 0.13]. However, as illustrated in Figure 1, women demonstrated shorter fixations [*t*(68) = 3.10, *p* = 0.003, Cohen’s *d* = 0.74] but larger duration of saccades [*t*(68) = 2.87, *p* = 0.005, Cohen’s *d* = 0.69] and larger average [*t*(68) = 2.65, *p* = 0.01, Cohen’s *d* = 0.61] and total [*t*(68) = 3.53, *p* = 0.001, Cohen’s *d* = 0.84] amplitude of saccades compared to men.

### 3.2. Higher Autobiographical Specificity in Women and Men

The analyses demonstrated higher autobiographical specificity in women (*M* = 3.50, *SD* = 0.44) than in men (*M* = 3.12, *SD* = 0.62) [*t*(68) = 2.89, *p* = 0.005, Cohen’s *d* = 0.69].

### 3.3. Relationship between Autobiographical Specificity and Eye Movement

The covariate analysis demonstrated no significant gender differences regarding fixation duration [*F*(1, 67) = 0.41, *p* = 0.52, η_p_^2^ = 0.092], saccade duration [*F*(1, 67) = 0.33, *p* = 0.57, η_p_^2^ = 0.073], or average [*F*(1, 67) = 0.88, *p* = 0.35, η_p_^2^ = 0.15] or total [*F*(1, 67) = 0.76, *p* = 0.39, η_p_^2^ = 0.10] amplitude of saccades after controlling for autobiographical specificity. Thus, the significant gender differences in eye movement disappeared after controlling for autobiographical specificity. For convenience, we carried out a covariance analysis for the remaining eye movement variable and found no significant gender differences regarding the number of fixations [*F*(1, 67) = 0.01, *p* = 0.92, η_p_^2^ = 0.052] or saccades [*F*(1, 67) = 0.42, *p* = 0.52, η_p_^2^ = 0.10].

### 3.4. Additional Analyses

We further carried out covariance analysis for verbal episodic memory (i.e., performance on the task of Grober and Buschke). The analysis demonstrated that after controlling for verbal episodic memory, women demonstrated significantly shorter fixation [*F*(1, 67) = 10.72, *p* = 0.002, η_p_^2^ = 0.90] but larger duration [*F*(1, 67) = 5.61, *p* = 0.021, η_p_^2^ = 0.65] and larger average [*F*(1, 67) = 0.49, *p* = 0.49, η_p_^2^ = 0.11] and total [*F*(1, 67) = 8.42, *p* = 0.005, η_p_^2^ = 0.82] amplitude of saccades than men. Thus, gender differences in eye movement were not influenced by verbal episodic memory. For convenience, we carried out covariance analysis for the two remaining eye movement variables and found no significant effect of gender regarding the number of fixations [*F*(1, 67) = 0.04, *p* = 0.84, η_p_^2^ = 0.055] or saccades [*F*(1, 67) = 0.79, *p* = 0.38, η_p_^2^ = 0.14].

## 4. Discussion

We, for the first time, investigated gender differences in eye movement during autobiographical retrieval. While no significant gender differences were found regarding the numbers of fixations and saccades, shorter fixations and larger saccades were observed in women than in men.

The shorter fixations and larger saccades, as activated by autobiographical retrieval in women compared to men, demonstrated that women tend to generate a larger gaze scan compared to men, who tend to fixate more, when retrieving autobiographical memories. The large gaze in women may mirror an exploratory gaze strategy involving the creation of larger visual scenes compared to men, who generate narrower visual scenes but with longer fixations. The large gaze in women, as observed in the current study, aligns with research demonstrating that women show high explorative gaze behavior, expressed by large saccades, when exploring scenes [25,26,28,29]. In a similar vein, Mercer Moss, Baddeley [36] demonstrated that the fixation distributions of women are larger than those of men when exploring pictures of art pieces and social interactions. Further, the explorative gaze in women as observed in our study aligns with hypothesis of selective information processing [37,38]. This hypothesis proposes that, compared to women, men are more selective processors as they consider only a subset of the available information when visually processing an environment. Taken together, the larger saccades during autobiographical retrieval may mirror the exploratory gaze behavior in women.

An alternative, but complementary, account for the significant gender differences in eye movement is autobiographical specificity. As demonstrated by our covariate analysis, the significant gender differences regarding fixation and saccade duration and amplitude were not present after controlling for autobiographical specificity. Thus, the shorter fixations and larger saccades in women than in men can be attributed to the women’s ability to retrieve detailed autobiographical memories. More specifically, we suggest that the exploratory gaze behavior in women may mirror the retrieval of a higher number of autobiographical details compared to men. Note that the higher autobiographical specificity, as observed in women in our study, mirrors previous research demonstrating that, compared to women, men retrieve events with less richness of details, such as how the event occurred [39,40] and even regarding factual details, such as where the event occurred [41,42] or to whom a message was previously told [1]. Taken together, the exploratory gaze behavior in women may mirror the richness of spatiotemporal/factual details during autobiographical retrieval.

Besides autobiographical specificity, the large saccades in women can be influenced by phenomenological characteristics of retrieval, especially by the vividness of mental imagery. Mental imagery refers to the ability to generate and manipulate mental images of the retrieved memories [43], and this ability is closely linked to the subjective experience of autobiographical memory: the more vivid the constructed image, the stronger the subjective experience [44,45]. Mental imagery is also closely associated with eye movement during autobiographical retrieval [16,17,18,21]. The role of visual imagery was shown by Lenoble, Janssen [20], who invited participants to remember autobiographical events while fixating on a cross in the center of a screen or while exploring the screen without constraints. This study showed that memories retrieved in the free-gaze condition triggered higher mental imagery than did those retrieved in the exploration-without-constraints condition. Thus, compared to those in men, the shorter fixations in women, as observed in the present study, can be associated with higher visual imagery. Besides mental imagery, the shorter fixations in women can be attributed to stronger emotional experience compared to that in men. This hypothesis is supported by a study showing that women express more affect than men when retrieving autobiographical memories [41]. While appealing, this assumption should be interpreted with some caution because our study design did not involve an assessment of emotion or mental imagery, or even the phenomenological characteristics of retrieval in general. This is a limitation of our study design that should be further investigated in future work.

Unlike the effect of autobiographical specificity, gender differences in eye movement in our study were independent of verbal episodic memory. As demonstrated by the covariance analysis, women demonstrated significantly shorter fixation and larger saccades even after controlling for scores on the task of Grober and Buschke. The lack of effects of verbal episodic memory on eye movement in our study can be attributed to the fact that we assessed verbal episodic memory using a general word list, while autobiographical specificity was assessed regarding the ability to retrieve personal episodic details. Hence, the short fixations but large saccades in women can be attributed to the ability to retrieve detailed personal information (i.e., where and when a personal event has occurred), rather than to the ability to retrieve general/semantic verbal information. However, the high verbal episodic memory in women, as observed on Grober and Buschke’s test in our study, mirrors research demonstrating high item memory in women [46,47], especially regarding the retention of verbal information [48].

### Strengths and Limitations

One limitation of the present study may be the lack of assessment of phenomenological characteristics of memories, such as whether differences in eye movement would be observed between men and women regarding the emotional characteristics of memories. Future research may thus assess gender differences for these characteristics. Another suggestion would be the assessment of gender differences regarding the temporal distribution of memories in light of research demonstrating how eye movement may differ between recent and old memories [19]. However, regardless of these limitations, our study has the merit to shed light on gender differences in eye movement during autobiographical retrieval in general.

## 5. Conclusions

To summarize, one behavior often made in everyday life by humans is gaze. Gaze can be triggered by external or even internal stimuli, as demonstrated by research showing how the retrieval of autobiographical memory triggers gaze. Our study expands this research by shedding light on gender differences. In fact, women exhibited larger saccades and smaller fixation than men, reflecting a larger gaze scan, which could reflect an exploratory gaze strategy. Interestingly, these eye movement pattern differences related to gender were not observed when autobiographical specificity was controlled for. This suggests that some cognitive processes (e.g., visual imagery, detail richness) underlying autobiographical specificity could account for the larger gaze scan pattern observed in women. Thus, this study contributes not only to the emergent research on eye movement during autobiographical memory, but also to the well-established research on cognitive and physiological processes related to gender differences.

## Figures and Tables

**Table 1:** Characteristics of eye movements under both conditions.

	Women	Men	
Fixation count per min	89.83 (24.21)	94.43 (31.48)	*p* = 0.50
Fixation duration in ms	565.15 (216.66)	766.77 (317.83)	*p* = 0.003
Saccade count per min	94.51 (23.65)	91.00 (28.75)	*p* = 0.58
Saccade duration in ms	39.75 (11.95)	31.29 (12.67)	*p* = 0.005
Average amplitude of saccades	7.09 (2.59)	9.63 (5.05)	*p* = 0.01
Total amplitude of saccades	1320.57 (620.65)	913.77 (281.61)	*p* = 0.001

Note. Standard deviations are in parentheses.

## Data Availability

Raw data are available upon request to the corresponding author.
